# On the Microstructural, Mechanical and Tribological Properties of Mo-Se-C Coatings and Their Potential for Friction Reduction against Rubber

**DOI:** 10.3390/ma14061336

**Published:** 2021-03-10

**Authors:** Jorge Caessa, Todor Vuchkov, Talha Bin Yaqub, Albano Cavaleiro

**Affiliations:** 1 Department of Mechanical Engineering, CEMMPRE, University of Coimbra, Rua Luís Reis dos Santos, 3030-788 Coimbra, Portugal; jorge.miguel.caessa.sousa@gmail.com (J.C.); talha.yaqub@gmail.com (T.B.Y.); albano.cavaleiro@dem.uc.pt (A.C.); 2 LED & MAT–IPN—Laboratory for Wear, Testing and Materials, Instituto Pedro Nunes, Rua Pedro Nunes, 3030-199 Coimbra, Portugal

**Keywords:** transition metal dichalcogenides, rubber tribology, magnetron sputtering, Mo-Se-C coating, solid lubricant, “chameleon” nanocomposite

## Abstract

Friction and wear contribute to high energetic losses that reduce the efficiency of mechanical systems. However, carbon alloyed transition metal dichalcogenide (TMD-C) coatings possess low friction coefficients in diverse environments and can self-adapt to various sliding conditions. Hence, in this investigation, a semi-industrial magnetron sputtering device, operated in direct current mode (DC), is utilized to deposit several molybdenum-selenium-carbon (Mo-Se-C) coatings with a carbon content up to 60 atomic % (at. %). Then, the carbon content influence on the final properties of the films is analysed using several structural, mechanical and tribological characterization techniques. With an increasing carbon content in the Mo-Se-C films, lower Se/Mo ratio, porosity and roughness appeared, while the hardness and compactness increased. Pin-on-disk (POD) experiments performed in humid air disclosed that the Mo-Se-C vs. nitrile butadiene rubber (NBR) friction is higher than Mo-Se-C vs. steel friction, and the coefficient of friction (CoF) is higher at 25 °C than at 200 °C, for both steel and NBR countersurfaces. In terms of wear, the Mo-Se-C coatings with 51 at. % C showed the lowest specific wear rates of all carbon content films when sliding against steel. The study shows the potential of TMD-based coatings for friction and wear reduction sliding against rubber.

## 1. Introduction

Friction and wear cause energetic losses that decrease the efficiency of mechanical systems [1]. The world’s estimated energy consumption emanating from tribological contacts due to wear and friction phenomena is approximately 23% [2]. Lubrication is an effective way to reduce energy wastage. Liquid lubricants form a protective film between interacting surfaces preventing solid/solid contact, and limiting the heat generated by friction. However, in many cases, the presence of liquid is neither possible nor recommended [3]. For example, the wide use of liquid lubricants is strictly limited at low pressures or vacuum, in corrosive environments, at low or elevated temperatures, and in situations where lubricant replacement is complicated [4]. Rough calculations estimated that, if the world took advantage of the available technologies targeted at reducing friction and wear, the expected decrease in energy loss due to tribological contacts would be approximately 40% in the short term. Globally, this would correspond to 1.4% of the gross domestic product (GDP) annually and a reduction in CO_2_ emissions by up to 1460 MtCO_2_ [2]. Therefore, scientific investigations on solid lubricants, which can replace conventional liquids where possible, have accelerated in recent decades. Solid lubricants currently under research are required to maintain a low coefficient of friction (CoF) and a low wear rate in a wide range of environmental conditions [1]. Transition metal dichalcogenides (TMDs) fulfil this condition to some extent. TMDs include sulphides, selenides and tellurides of transition metals (Mo, W or Nb) [5]. When in the hexagonal form, they are among the best alternatives of low friction coatings in dry air and vacuum [6]. The structure of TMDs consists of a stack of layers. The transition metal atoms occupy a middle layer, which is sandwiched above and beneath by layers of chalcogens. Since transition metals and chalcogens attract through strong covalent bonds, while TMD sandwiches bond through weak van der Waals forces, TMDs have an anisotropic behaviour [5,7]. Hence, during sliding, easier slip occurs between lamellae. Despite their potential, TMDs have some limitations, including low adhesion on standard substrate materials (e.g., steel), high porosity and very low hardness and load-bearing capacity, compared to competitive coatings such as diamond-like carbon (DLC) [6,7]. Adhesion can improve if a thin metallic interlayer, commonly Ti or Cr, is deposited between the substrate and TMD. Nonetheless, porosity, hardness and load-bearing capacity are problematic. First attempts to alloy TMDs aimed to restrain their fundamental drawbacks. Metallic and non-metallic elements and compounds were all added to TMDs. Metal dopants (Ti, Al, Au, Pb, Ni, Cr and others) showed some improvements, with Ti being the most successful from a commercial point of view. On the other hand, non-metallic compounds, such as ZnO, Sb_2_O_3_ or PbO, did not show tangible benefits [6]. However, a promising solution, consisting of the co-deposition of TMDs and carbon, was proposed and presented by Voevodin et al. [8]. With this solution, the tribological behaviour of TMDs in dry air and vacuum was agglutinated with that of diamond-like carbon (DLC) in humid air. So, the scientists deposited W-S-C coatings by magnetron-assisted pulsed laser deposition (MSPLD) and laser ablation. Indeed, the films revealed a “chameleon behaviour”, adapting the low friction properties according to the environment they were in (dry air or humid air). Follow-up studies from Cavaleiro et al. [9,10] showed that tungsten-sulphur-carbon (W-S-C) coatings deposited by magnetron sputtering exhibited the same adaptive features. However, W-S-C systems presented relatively high wear and CoF in humid environments. Thus, molybdenum diselenide substituted tungsten disulphide, because it displayed low friction almost independently from the air humidity, contrary to the other TMDs [5]. Polcar et al. [7,11,12,13] studied in detail Mo-Se-C deposited by radio frequency (RF) magnetron sputtering of a carbon target with MoSe_2_ pellets placed in the erosion zone. Despite the functional temperature limit of 250 °C, the outcomes were positive since the Mo-Se-C films (MoSe_2_ nanocrystals enrobed in a C matrix) extended the low CoF to humid air. The responsible phase for low friction was a MoSe_2_ tribolayer formed in the contact region, which was reoriented parallel to the coating surface, even at small depths beneath it. Carbon contributed majorly to bulk properties such as the density, hardness and load-bearing capacity [6,7]. However, a problem persisted. The deposition procedure carried out by Polcar et al. was not suited for upscaling at an industrial level due to working with radio frequency (RF) power supplies and utilizing composite targets. To overcome this issue, Mo-Se-C coatings were deposited by closed-field unbalanced magnetron sputtering, with direct current (DC) power supplies and individual targets of MoSe_2_ and carbon, firstly in a laboratory-scale deposition unit and, later, in semi-industrial deposition equipment [14,15]. The coatings provided outstandingly low wear and friction when sliding against steel-based material in different environments. The deposition of Mo-Se-C coatings in semi-industrial conditions is rather scarce. In a recent study on Mo-Se-C coatings [15] in our research group, it was shown that a concurrent bombardment (i.e., application of substrate bias) of the growing film during deposition improved the hardness and morphology of the films without hindering the lubricity. Nevertheless, that study was limited to an optimization for coatings with a carbon content of ~ 50% at. Consequently, in this investigation, the authors used the same semi-industrial deposition apparatus to deposit Mo-Se-C coatings with carbon content up to ~ 60 at. % of C. The additional novelty of this work was still not revealed: can a Mo-Se-C positively affect the tribological efficiency of metal vs. rubber sliding? Metal vs. rubber frictional contact occurs in several applications, such as sealing and polymer moulding processes [16,17]. For instance, during tire moulding and mould release, friction and adhesion phenomena lead to considerable rubber losses. Despite an already existing solution to solve the tire moulding problems, the chemical release agents, this solution is not permanent. The number of commercial release products available is evidence that there is no unanimous solution to this problem yet. Hence, the deposition of a Mo-Se-C film on the mould surface might provide a longer-lasting lubricating effect and much higher effectiveness, avoiding all the time currently spent to lubricate the sliding regions in the moulding process [3,17,18]. Potentially, Mo-Se-C coatings could be a good fit, economically, environmentally and performance-wise, in the polymer moulding market. Therefore, this article describes the first results on alloyed transition metal dichalcogenide coatings sliding against rubber. Nitrile butadiene rubber (NBR) is tested tribologically against several Mo-Se-C films, at both 25 and 200 °C. For comparative purposes, the tribological experiments are also performed against a steel-based counterbody. With this investigation, the authors aim to provide information and discuss the properties of Mo-Se-C deposited in semi-industrial conditions with various carbon contents. Furthermore, the study also focuses on the tribological properties of the coatings, especially during sliding against rubber.

## 2. Materials and Methods

### 2.1. Materials Utilized

Polished AISI M2 steel (the composition is shown in Table 1) was utilized as the substrate material, with a hardness of ~9 GPa, a diameter of 24 mm and a thickness of 8 mm. To polish, SiC papers (from P120 grit size) followed by magnetic-backed cloths (until grit size of 3 μm, with adequate diamond suspension) were used. The achieved surface roughness of the samples was R_a_ < 20 nm. Si wafers were also employed as substrates for posterior grazing incidence X-ray diffraction (GIXRD) analysis. Both M2 steel specimens and Si wafers were ultrasonically cleaned for 10 min in acetone and ethanol and dried with hot air before placement in the chamber. Then, the substrates were positioned in rotating carriers in the deposition chamber.

### 2.2. Deposition Method

#### 2.2.1. Equipment and Target-to-Substrate Distance

In this investigation, the deposition procedure was kept similar to one recently utilized by Vuchkov et al. [15]. Teer UDP 650/4 (Teer Coatings Ltd., Droitwich, UK) closed-field unbalanced magnetron sputtering equipment, of small industrial-scale (with a volume of 275 dm^3^), was used. Four planar targets (380 × 175 × 8 mm), vertically aligned on the chamber walls with a 90° angle between them, were utilized. The target configuration was shown in our previous study [15]. The targets were 1 Cr target, 1 MoSe_2_ and 2 graphite targets, all with 99.9% purity. The utilization of two graphite targets was due to the lower sputtering yield of carbon relative to the other materials. Meanwhile, the chosen target-to-substrate distance was 15 cm. This distance affords superior tribological performance at high temperatures, as demonstrated in the above-cited study.

#### 2.2.2. Deposition Procedure

The first stage of deposition was cleaning. Covering shields were applied to each target to prevent cross-contamination from reaching the substrates. Then, targets and substrates were sputter cleaned. During cleaning, pulsed bias voltages (Advanced Energy Pinnacle plus, Fort Collins, CO, USA, 600 V, 250 kHz reverse time of 1.6 μs) were applied only to the substrates. The targets were connected to DC power supplies (Advanced Energy Pinnacle, Fort Collins, CO, USA). The cleaning time was 20 min for each pair of targets (e.g., MoSe_2_ and opposite graphite, and Cr and its opposite graphite) and 40 min for the substrates. Sequentially, the deposition process was undertaken. A current control mode was employed to fix the total current inputted to the targets. The global time of deposition was 120 min. The earliest 10 min were specifically for depositing an interlayer, with 7.5 mA/cm^2^ applied to the Cr target. Throughout the 10 min, the burial of a gradient layer happened. During the gradient layer deposition, an increase in both carbon and MoSe_2_ target currents occurred in alignment with a decrease in the Cr target current. Each final deposition step encompassed 100 min, and one out of five distinct carbon content coatings (pure MoSe_2_, 24 at. % of C, 33 at. % of C, 51 at. % of C, 60 at. % of C) was deposited. Throughout the deposition process, the chamber pressure was 0.4 Pa (Ar atmosphere), and a DC substrate bias of −50 V was applied to the substrates. Herein, the coatings will be addressed as MoSeCx, where x is the carbon content (e.g., the coating with 51 at. % of carbon will be referred to as MoSeC51). The coating deposited without applied current to the graphite targets will be referred to as MoSe. However, residual carbon contents of ~6 at. % were present in the MoSe film, which likely came from cross-contamination originating from the graphite targets. 

### 2.3. Characterization Techniques

**Figure 1:**
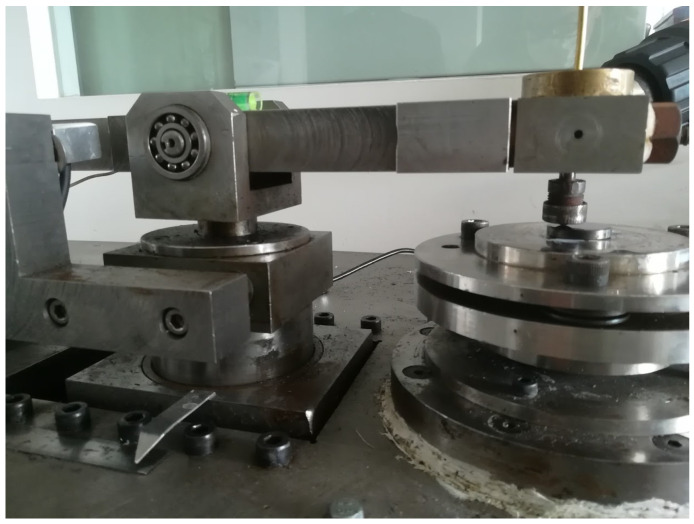
Customized pin-on-disk (POD) machine utilized for tribological testing.

A wavelength dispersive spectroscopy (WDS) detector (Oxford Instruments, High Wycomb, UK) attached to a field emission scanning electron microscope (FESEM) (Zeiss Merlin, Oberkochen, Germany) was used for analysis of the composition. The latter instrument allowed us to assess the thickness and morphology. Atomic force microscopy (AFM) (Bruker Innova, Billerica, MA, USA) allowed us to analyse the surface morphology of the films. AFM measurements were taken with a Si tip (AppNano Acta, Mountain View, CA, USA) with radius <10 nm, operating in tapping mode. Probed areas had a size of 5 by 5 µm. A cantilever with a resonance frequency of 200–400 kHz and a spring constant of 13–77 N/m was used. Processing software Gwyddion [19] also enabled us to analyse the topography and evaluate the roughness. GIXRD (PANalytical X’pert MRD, Almelo, The Netherlands) was the technique utilized for investigating the crystal structure. Copper Kα radiation (U = 40 kV, I = 45 mA) hit the specimens at an incident angle of 3° and scanned in a 2θ range of 5°–90°. A scratch test apparatus (Revetest, CSEM Instruments, Neuchâtel, Switzerland) evaluated the adhesion of the coatings. It made use of a conical diamond stylus with a tip radius of 0.2 mm, drawn across the samples at a constant velocity of 10 mm/min and a varying load of 2–80 N, with a loading rate of 10 N/mm. Post facto, an optical microscope (Leica DM4000 LED, Wetzlar, Germany) inspected the failure modes. Indentations were carried with a diamond cone Rockwell C type indenter too, at a load of 1471 N, to confirm the scratch test results. The degree of adhesion of the films was verified, with the verein deustcher ingenieure (VDI) guideline 3198 [20] as a base. A nanoindentation device (Nanotest, Micro Materials Ltd., Wrexham, UK) equipped with a Berkovich diamond pyramid indenter was used to check the hardness and reduced elastic modulus of the specimens. In total, 16 nanoindentation measurements were carried, at ambient temperature, with loads of 1 mN for MoSe and of 3 mN for the remaining coatings. Selection of indentation loads had the objective to maintain the indentation depths smaller than 10% of the coating thickness. The conduction of data analysis used Oliver and Pharr’s method [21]. As for tribological characterization techniques, a customized pin-on-disk (POD) machine (shown in Figure 1) performed experiments in 20 distinct conditions: other than the diversity of carbon contents attributed to each group of coatings (MoSe, MoSeC24, MoSeC33, MoSeC51, MoSeC60), the tests also differed in temperature (25 or 200 °C), and in the materials sliding against the films (NBR or DIN-Deutsches Institut für Normung 100Cr6 steel balls). For each situation, at least two tests were performed. Other conditions were constant throughout the experiments: the sliding velocity of 0.05 m/s, the load of 10 N and the relative humidity (RH) of approximately 40%. 

Finally, a 3D optical profilometer (Alicona Infinite Focus, Raaba, Austria) made it possible to calculate the specific wear rate, which is related to the wear performance of the coatings, under each test condition. The 3D optical profilometer allowed us to measure the sectional area removed from the POD experimental coatings due to wear. The worn cross-sectional area was estimated by taking the average of 4 separated profiles across the worn track. The removed area was then multiplied by the wear track circumference to calculate the wear volume.

The specific wear rate (SWR) formulates as:

(1) $$W_{r}\;=\;\frac{V_{w}}{F\cdot d}$$

where $W_{r}$ is the specific wear rate coefficient in mm^3^/Nm, $V_{w}$ is the already described worn volume in mm^3^, F is the perpendicularly applied force in N and d is the sliding distance in m. Post-test scanning electron microscopy and energy dispersive spectroscopy (SEM/EDS) analysis of the wear tracks was also performed. This analysis was focused on the tribological tests against rubber surfaces. The rubber counterbodies were presputtered with gold before the SEM/EDS analysis.

## 3. Results and Discussion

### 3.1. Chemical Composition

**Figure 2:**
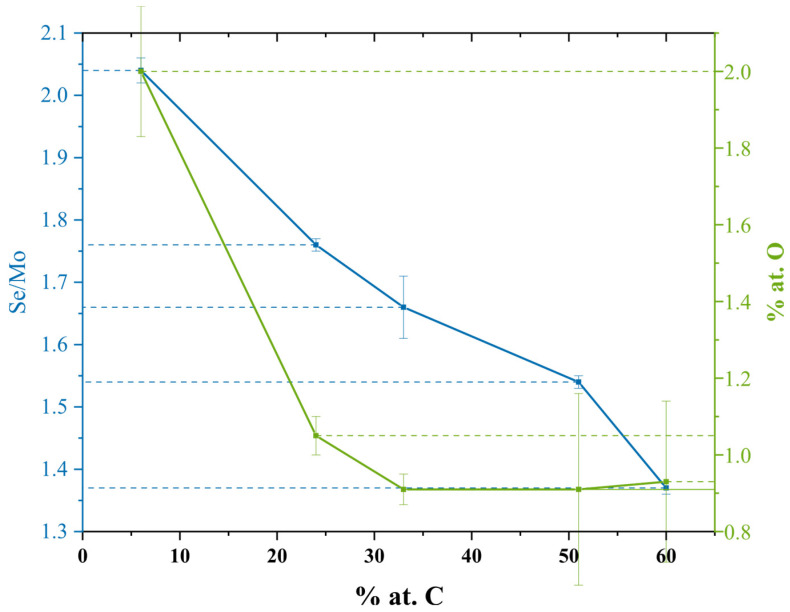
Se/Mo ratio and oxygen content for the varied carbon content samples.

WDS permitted us to obtain the chemical composition of the deposited coatings. Figure 2 displays a summary of the outcomes.

The oxygen content was almost negligible for every film, reaching a maximum of about 2 at. % of O for MoSe. Not surprisingly, MoSe exhibited the highest contamination, probably due to its very porous morphology, which is more prone to oxidation. Afterwards, with the increase in carbon content, the oxygen content decreased steeply up to the 33 at. % of C. For samples with superior carbon contents, the oxygen presence seemed to stabilize. This event was likely related to an approximate plateau attained regarding the porosity and compactness of the coatings. The most probable contamination sources were the residual oxygen in either the deposition chamber or the MoSe_2_ target [15]. The Se/Mo ratio followed a slightly different trend. It almost linearly diminished with the increase in carbon content in the Mo-Se-C specimens. The high current applied to the graphite targets likely originated from an increased Ar ionization in the substrate vicinity. Then, substrate bias application probably caused higher bombardment on the growing films, resulting in accelerated preferential re-sputtering of the lighter Se atoms. The reduced Se/Mo ratio for the samples with high carbon content can thus be ascribed to this superior level of bombardment on the growing film with energetic species [22]. A reduction in the Se/Mo ratio generally foments a worse sliding scenario by impairing the crystallinity of the MoSe_2_ phase.

### 3.2. Morphology and Topography

**Figure 3:**
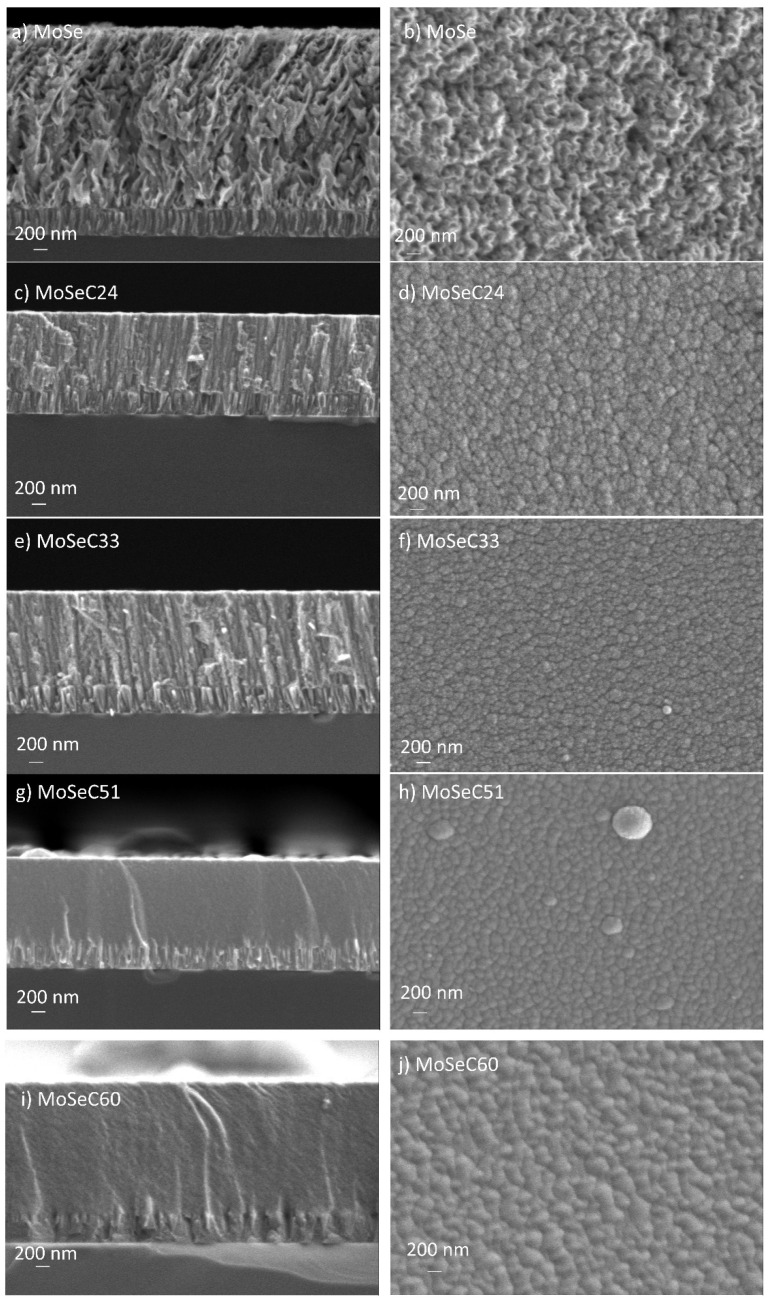
Cross section (CS) and surface morphology (SM) of the Mo-Se-C coatings obtained via SEM: (**a**) CS of MoSe; (**b**) SM of MoSe; (**c**) CS of MoSeC24; (**d**) SM of MoSeC24; (**e**) CS of MoSeC33; (**f**) SM of MoSeC33; (**g**) CS of MoSeC51; (**h**) SM of MoSeC51; (**i**) CS of MoSeC60; (**j**) SM of MoSeC60.

SEM imaging enabled the evaluation of the cross-section, surface morphology and thickness of the coatings. Depictions of the cross-section and surface morphology are present in Figure 3.

SEM images confirmed a cross-section with columnar structures for the MoSe. These were associated with relevant porosity between the columns, as commonly arises for sputtered TMD films [22]. The surface morphology of the MoSe coating showed features akin to sponges. Hence, the film was highly porous, as observed in previous works of Yaqub et al. [14,23]. With carbon doping, the columnar cross-section gradually vanished. Even though the films with C content lower than 33 at. % still manifested a degree of columnar structure, they presented a much denser morphology, with fewer voids between columns, as the carbon content increased. Additionally, the cross-section became more homogeneous. The higher deposition rate for the unalloyed MoSe coatings, despite the additional sputtering promoted on the graphite targets in the carbon alloyed coatings, was further evidence of densification [15]. SEM surface morphology images revealed a granular cauliflower-like appearance for the films with carbon presence (MoSeC24 and MoSeC33), whilst a dome-grain alike morphology was observed for the higher carbon content films (MoSeC51 and MoSeC60). The changes in surface morphology with the increasing carbon content were probably due to an increase in surface mobility. In essence, the higher current applied to the carbon targets likely manifested in an elevated temperature at the substrate, due to an increased bombardment of the growing films with Ar^+^ ions. In this context, the coatings with higher carbon content were likely deposited under a condition of increased adatom mobility, ultimately leading to an increase in the density of the films [24]. Compared to a previous work where Mo-Se-C films were deposited in smaller scale laboratory equipment [23], the morphological features for the MoSeC24 and MoSeC33 indicated a reduced density. For example, the coating with 27 at. % of carbon in that study had a denser, featureless cross-sectional appearance. This dissimilarity is very likely due to the different sputtering arrangements and sheds light on the distinctions encountered during upscaling of TMD-based thin films developed in smaller, laboratory-scale sputtering equipment. On the one hand, the above-mentioned laboratory-scale equipment, which provides shorter target-to-substrate distances, has a confocal configuration with a continuous arrival of the sputtered species from three targets, simultaneously. On the other hand, in the semi-industrial arrangement, the substrates are periodically exposed to a flux of sputtered species, which probably increases the atomic shadowing effects, resulting in columnar morphologies with reduced densities. Indeed, the coatings with the highest carbon content showed a featureless cross-sectional appearance, in agreement with previous studies [14]. The atomic shadowing effects, in this case, were reduced due to an increased concurrent bombardment of the growing film with Ar^+^ ions as well a reduced content of the inherently porous MoSe_2_ phase. Table 2 represents the measured thickness and roughness of the coatings.

Until MoSeC33, a decrease in the thickness took place with increasing carbon. A reverse trend materialized for the MoSeC51 and MoSeC60 films. The unalike trends likely occurred due to a compromise between the increase in the compactness, for low C contents addition, and the larger deposition rates, with increasing current applied to the graphite targets, for the higher carbon content coatings. The average roughness of about 26 nm found for MoSe was quite similar to that investigated by Vuchkov et al. for a similar deposition procedure [15]. The carbon-doped coatings were smoother, with average values as small as 4.5 nm for MoSeC60. The observed roughness trends are in accordance with the morphological features observed during SEM analysis. The coatings with more compact featureless appearance have lower roughness. This is due to the aforementioned reduced atomic shadowing effects observed for the coatings with higher carbon content.

### 3.3. Crystal Structure

**Figure 4:**
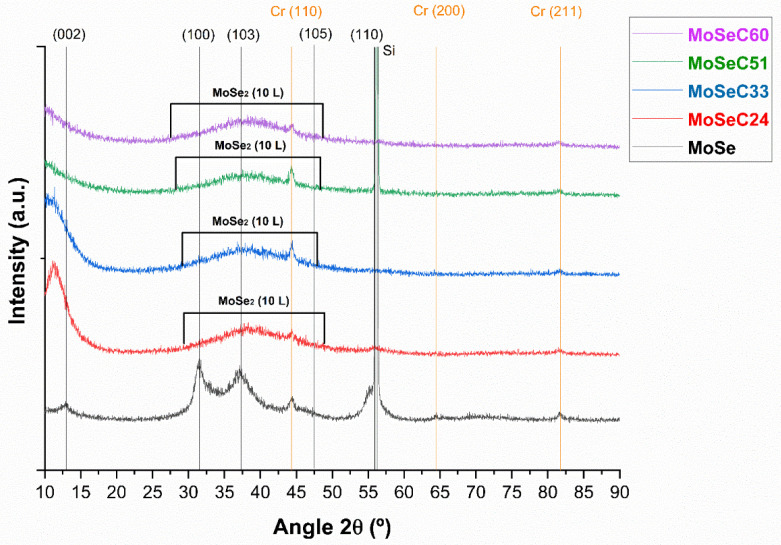
GIXRD diffractograms for all the Mo-Se-C samples.

GIXRD permitted us to examine the crystallography of the coatings. Figure 4 shows the plotted outcomes.

The GIXRD diffractogram of MoSe presents several MoSe_2_ orientations with the strongest diffractions occurring for plane orientations of basal (002) at 2θ ~ 13°, of (100) at 2θ ~ 31.5°, of (103) at 2θ ~ 37.5°, of (105) at 2θ ~ 47.5° and of (110) at 2θ ~ 56° (Reference ICCD card No. 01-0771715). Peaks stemming from the Cr interlayers at 2θ ~ 44.5°, 2θ ~ 64.5° and 2θ ~ 82° (Reference ICCD card No. 01-0851335) were also observed. Not surprisingly, GIXRD patterns displayed a loss of crystallinity of the coatings as carbon content increased. Hence, the MoSe was the only undoubtedly crystalline film. When a GIXRD peak is broader, the coating is tendentially more amorphous [13]. The concept referred to lastly was verified along the 2θ span of ~30°–50° in the carbon-doped coatings. A turbostratic stacking of the MoSe_2_ (10 L) planes, with L taking the values of 1, 2, 3, …, was identified, as often happens in sputtered alloyed TMD coatings [1,25]. The turbostratic stacking fundamentally signifies that the usual ABAB hexagonal stacking might have suffered translations or rotations relative to its most ordered structure [26]. An enhancement of carbon content in the samples additionally translated in a shift of the (002) peak position to reduced 2θ angles. The cause of this was an increase in the interplanar distances. The former roots diminished van der Waals adhesive forces between TMD basal planes and can facilitate the easy shearing properties of MoSe_2_ [23]. Another trend observed was the presence of smaller MoSe_2_ crystallites, as suggested by the broader (002) peaks in the coatings containing carbon. Thus, carbon must disturb the growth of the MoSe_2_ crystals, and induce the thickness to decrease, even though the interplanar distances follow the inverse path. As known, the mentioned (002) orientation correlates to easier sliding by the MoSe_2_ phase and higher protection against oxidation [6]. It represents the basal planes that are nearly parallel to the substrate and have a pivotal role in the tribological properties. On the other hand, the (100) orientation stands for basal planes perpendicular to the substrates (edge orientation). These latter coatings require a reorientation of the MoSe₂ to provide low friction [27]. Therefore, the coatings MoSeC24 and MoSeC33 should have a better frictional response due to the presence of MoSe_2_ crystals oriented nearly parallel to the coating surface.

### 3.4. Mechanical Properties

**Figure 5:**
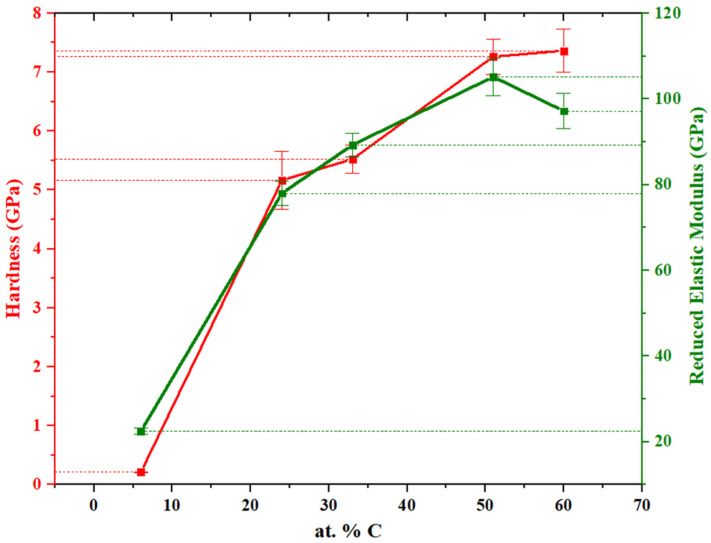
Results from nanoindentation experiments: hardness and reduced elastic modulus for each carbon content respectively.

Nanoindentation allowed us to ascertain, precisely, the hardness and the reduced elastic modulus, two fundamental mechanical properties. Figure 5 presents the average results for both properties.

The outcomes were quite similar for both properties. Measured values increased for superior carbon content films, although a slight nuance occurred for the elastic modulus in the elevated carbon content films. Owing to the low density and high porosity, the MoSe coating displays a very low hardness of about 0.25 GPa and an equally low reduced elastic modulus of about 21 GPa. By rising the carbon alloying in MoSeC, the compactness enhanced, which was reflected on a superior hardness and elastic modulus. In fact, for MoSeC24, the hardness increased to ~ 5 GPa, almost twenty-fold higher than the numbers observed for the MoSe coating. Moreover, the modulus increased considerably, from 21 to 79.5 GPa. Thus, low carbon additions immediately improved the mechanical performance by a large amount. MoSeC24 matched reasonably with MoSeC33. Analogously, the MoSeC51 and MoSeC60 showed similar mechanical properties within the error margins, except for the reduced elastic modulus. The continuous increment in the hardness with the increase in carbon was not totally verified in the modulus for the two highest carbon content coatings. Indeed, the more enhanced carbon content film exhibited greater hardness, about as high as 7.4 GPa. However, MoSeC51 presented the highest reduced elastic modulus of 105 GPa. Discrepancies were not immense. Yet, MoSeC60 only had 98 GPa of reduced modulus of elasticity, which can be considered a statistically relevant difference compared to the MoSeC51. Plausibly, the increase in hardness was due to the lower Se/Mo ratio, which fell significantly from 1.54 to 1.37 between MoSeC51 and MoSeC60 films. Thus, the literature claims that a higher Se/Mo ratio contributes to a lower hardness [28]. Nonetheless, an attained plateau in compactness might explain the modulus relationship amid MoSeC51 and MoSeC60. These coatings displayed similar oxygen contents and morphological features, agreeing with the hypothesis of a compactness plateau. Higher compactness of the same material usually translates into improved Young’s modulus because the last property measures a pressure divided by a strain [29]. At constant pressure, the strain produced on a more compact coating should generally be inferior. Since the two highest carbon content coatings showed close compactness, the modulus should remain comparable. Depending on the application, the most desirable combination of mechanical properties might translate into improved hardness and declined elastic modulus. H^3^/E*^2^ ratio, with E* being the reduced Young’s modulus, was larger for the MoSeC51 film, indicating higher fracture toughness and adhesion of the coating [30]. Additionally, the H/E* ratio, which relates to elastic strain at failure, also increased, meaning the wear resistance should improve [31]. Nanoindentation results are generally in agreement with the previous study on similar Mo-Se-C coatings [23]. It is noteworthy that although the coatings in the present study exhibited a slightly underdense columnar morphology, their hardness was quite similar to those obtained in laboratory-scale deposition conditions with similar compositions and superior morphological appearance.

### 3.5. Adhesion

A scratch test and Rockwell C indentation allowed us to assess adhesion. In the scratch experiment, a critical load (Lc) allows us to distinguish cohesive from adhesive failures. Cohesive failures are related to through-thickness cracks, i.e., chevron cracks. Instead, adhesive failures originate from compressive stresses that separate the coating from the substrate, i.e., spallation and buckling. Cohesive failures occur inside the coating region. Meanwhile, adhesive failures happen at the coating–substrate interface. Three different types of critical loads coexist. These include chevron cracks (Lc1), buckling and spallation (Lc2) and gross delamination of the coating on the full scratch width or Lc3. Even though the Lc (critical load) depends on several factors, such as the stylus-tip radius, the load rate or the coating thickness, the scratch experiment acts as a reasonable indicator on the coating–substrate adhesion and the toughness of the coating. Therefore, Table 3 presents an exposition of the outcomes from scratch experiments.

**Figure 6:**
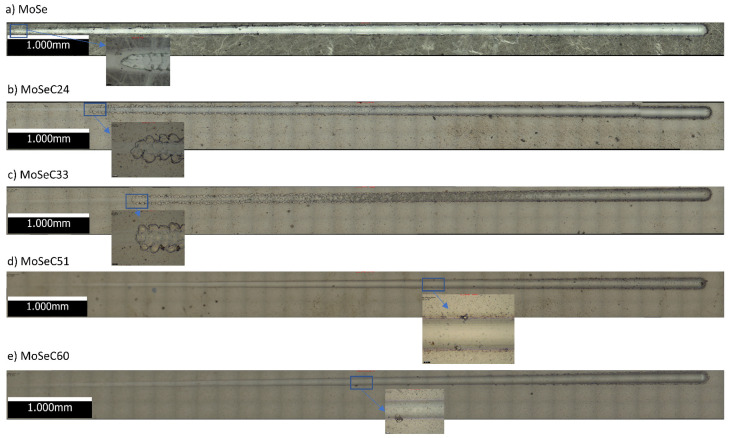
Scratch track micrographs: (**a**) MoSe coating; (**b**) MoSeC24; (**c**) MoSeC33; (**d**) MoSeC51; (**e**) MoSeC60.

Two neat trends transpired with the introduction of carbon in the films. First, the failure event associated with the coatings only occurred at greater loads. Second, it gradually moved from adhesive to cohesive. Thus, the films containing more carbon displayed better adhesion than the others. For the softer coatings (MoSe, MoSeC24 and MoSeC33), the only detected failure event was the adhesive Lc3. The failure became Lc2 in MoSeC51 coating, with spalling observed on the borders of the scratch scars. Finally, in the superior carbon content coating, the adhesion strength was undoubtedly the highest and unveiled the first cohesive Lc1 failure event, indicated by cracks on the border of the scratch track. The insets in Figure 6 show the onset of these failure events.

The MoSe coating revealed gross delamination. Because the coating held small hardness, it was ploughed away during the test, meaning it was unsatisfactory adhesion-wise. Then, MoSeC24 and MoSeC33 showed reasonable adhesion development, yet remaining very far from the intended values. Only for the MoSeC51 or upper carbon coatings, a substantial increase in adhesion emerged. No gross detachment of the film was detected, only some controlled spalling. Nonetheless, solely the MoSeC60 coating provided a guarantee of acceptance, with the onset of spalling occurring at high loads. Since it uses more readily available indenters, Rockwell C indentation is also an appealing adhesion assessment method at an industrial level. Thus, it provides a ready comparison of outcomes between investigated and industrially used coatings. In the Rockwell C indentation test, observed trends were identical to those found in scratch, as additionally shown in Table 3. Scales HF1 to HF6 were attributed to each coating, according to the VDI guideline 3198 [20]. The MoSe coating, allocated to the HF6 class, completely exposed the substrate in immediate regions to the indentation. Films with increased carbon revealed ameliorated adhesion. The latter was likely due to having higher hardness and compact featureless cross-sectional morphologies [15]. HF5 was the classification given to the MoSeC24 coating, as visible delamination occurred in the indentation vicinity. With slightly better adhesion, the MoSeC33 film uncovered large spalled areas, although it showed much less visible delamination and exposure of the substrate beneath the coating. The MoSeC51 and MoSeC60 exhibited a vast augmentation in adhesion, with only minor spalling and cracking around the indentation circumference. Both were considered HF2.

### 3.6. Tribological Properties

#### 3.6.1. Sliding against Steel (DIN 100Cr6) at Room Temperature (25 °C)

**Figure 7:**
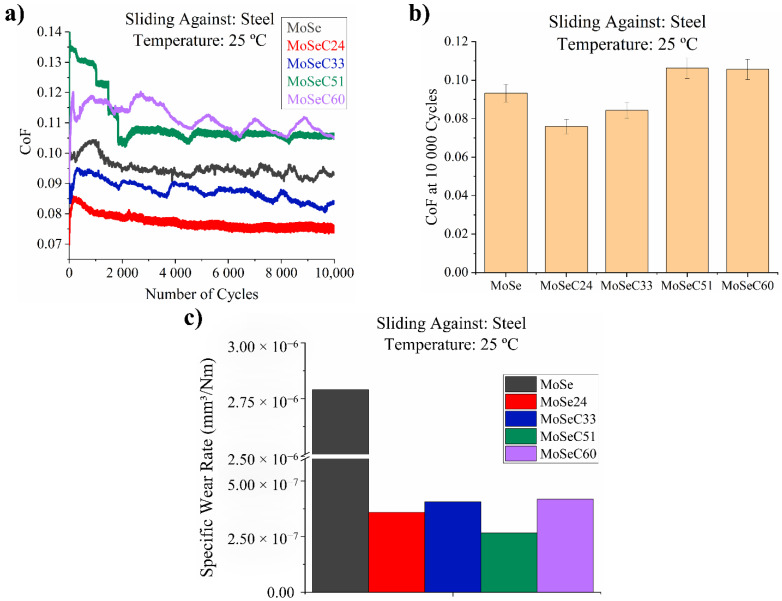
Results of the POD tests for coatings, performed against a steel countersurface, at 25 °C: (**a**) friction curves; (**b**) final coefficient of friction (CoF); (**c**) specific wear rates obtained via 3D optical profilometer.

The friction curves, the CoF and specific wear rates measured for the coatings sliding against a steel counterbody are presented in Figure 7.

The best frictional response was observed for the coating MoSeC24 (COF ~ 0.075), followed by the coating MoSeC33 (~0.08) and the MoSe (~0.09). The coatings with the highest carbon content (MoSeC51 and MoSeC60) attained CoF values of ~0.11. The outcomes possibly correlate with the morphological and microstructural features presented in the previous sections, namely, the frictional response was better for the MoSeC24 coating, which contains MoSe_2_ crystals aligned closely parallel to the sliding surface, as depicted from the GIXRD analysis (see Section 3.3). MoSeC24 additionally carries a larger amount of MoSe_2_ crystals, hence facilitating the lubricious TMD tribofilm formation. Instead, the films alloyed with extra carbon were more amorphous and displayed an increased CoF. Both coatings with the highest carbon content showed quite similar CoF, which is due to the fact that they are quite similar in terms of morphology and crystallinity. It is also plausible that the role of MoSe_2_ is diminished in this case, and the small change in chemical composition did not result in a significantly different CoF value. The MoSe coatings showed a frictional response lying between the results obtained for the carbon alloyed coatings with lower (MoSeC24 and MoSeC33) and augmented (MoSeC51 and MoSeC60) carbon content. The higher CoF observed for the MoSe coating can be related to its exquisitely low hardness as compared to the steel counterbody. Due to the significant differences in hardness, increased ploughing likely appeared, a tribological process that usually raises the CoF according to the Bowden–Tabor model for metallic friction [32]. Although the carbon phase can also provide friction reduction due to graphitization, we believe that the main lubricious (i.e., low shear strength phase) is the MoSe_2_. Reduction in the CoF through graphitization can have a small role in the coatings with the highest carbon content. We base these statements on the trends observed for the alloyed MoSe coatings in addition to reports in the literature on similar coatings (see [12,33]). The role of carbon is generally towards the improvement of the hardness, providing enhanced load support.

In terms of wear, the carbon alloyed coatings achieved an improved wear resistance, with specific wear rate (SWR) values in the range of 2.5–5 × 10^−7^ mm^3^/Nm. An expected outlier in the wear resistance is the MoSe coating that presents an SWR of ~2.75 × 10^−6^ mm^3^/Nm. The lower wear resistance of the MoSe coating is due to its very low hardness.

Compared with some recent studies on similar coatings (see [34]), the frictional response of the present films (MoSeC24 and MoSeC33) is worse, as the measured CoF increased. Differences are probably due to the slightly inferior morphological appearance as well as the small compositional differences. Overall, the coatings revealed comparable wear resistance to those available for TMD-C films in the literature [14,15,34,35].

#### 3.6.2. Sliding against Steel (DIN 100Cr6) at High Temperature (200 °C)

**Figure 8:**
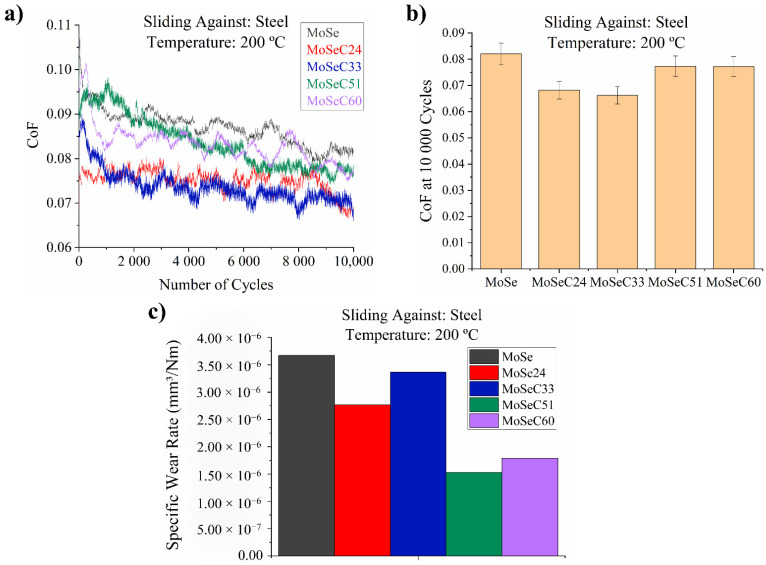
Results of the POD tests, performed against a steel countersurface, at 200 °C: (**a**) friction curves; (**b**) final CoF; (**c**) specific wear rates obtained via 3D optical profilometry.

Figure 8 presents the tribological performance of the coatings at elevated temperature.

A small reduction in the CoF was verified for the MoSe and low carbon content coatings (MoSeC24 and MoSeC33). However, for the films with increased carbon content, the friction reduction was even higher. The diminished CoF during testing at elevated temperature may be associated with the following mechanisms:

1. The atmosphere around the testing rig dried due to the elevated temperature, positively affecting the shear properties of the MoSe_2_ phase. It is well-known that lower relative humidity (RH) decreases the shear strength of the TMD materials, which increases their lubricity [36].
2. For the coatings with a higher degree of amorphous structure (e.g., MoSeC51 and MoSeC60), the additional thermal energy inputted to the contact probably facilitated the formation and reorientation of lubricious MoSe_2_ tribolayers, parallel to the sliding direction, between the coating and the steel ball surfaces [37].

In relation to the wear behaviour, a perceived result was the discrepancy between the wear rates at high and room temperature conditions. The wear of the coatings increased for 200 °C experiments, although in a shorter amount for MoSe. The increase in wear between the testing at room and at elevated temperature was almost one order of magnitude in some cases (e.g., ~3 × 10^−7^ mm^3^/Nm compared to ~3 × 10^−6^ for the MoSeC33 coating). This increase in wear can be related to the accelerated graphitization of the carbon phase. Since graphite exhibits inferior strength to the as-sputtered amorphous carbon, the worn particles glide towards the outside of the friction track and foment additional wear. Furthermore, the increase in testing temperature, in addition to the localized heating generated during sliding, can increase the oxidation of the MoSe_2_ phase, also contributing to increased wear.

#### 3.6.3. Sliding against Rubber (NBR) at Room Temperature (25 °C)

**Figure 9:**
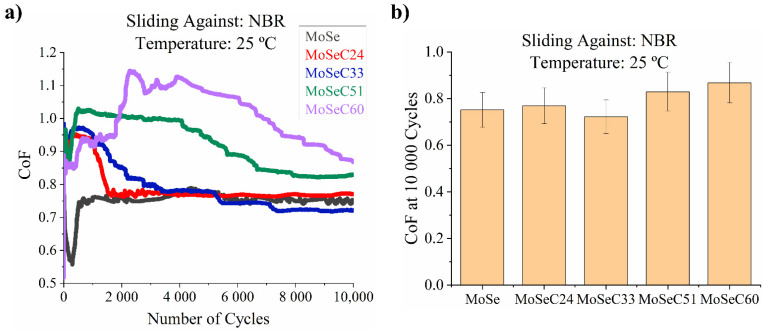
Results of the POD tests, performed against a nitrile butadiene rubber (NBR) countersurface, at 25 °C: (**a**) friction curves; (**b**) final CoF.

Figure 9 displays the results from the tribological tests of the coatings against NBR balls at 25 °C.

Compared to the outcomes achieved for the coatings tested against steel, the CoF is significantly higher when sliding against NBR rubber. The MoSe and the MoSeC coatings with low carbon content (MoSeC24 and MoSeC33) manifested the best results with CoF values in the range of 0.75–0.8. It is interesting to note that the MoSe coatings experienced a decrease in the CoF to ~0.6 in the initial 50 cycles, but eventually, the friction stabilized at ~0.75–0.8. The films with increased carbon content (MoSeC51 and MoSeC60) showed higher CoF with final values of ~0.85 and ~0.9, respectively. The trend observed indicates that the coatings richer in MoSe₂ can provide a friction reduction. It is also worth mentioning that the CoF of uncoated polished AISI M2 steel sliding against NBR in similar conditions has values which lay in the range of 1.1–1.4, in agreement with values presented in the literature [38,39].

**Figure 10:**
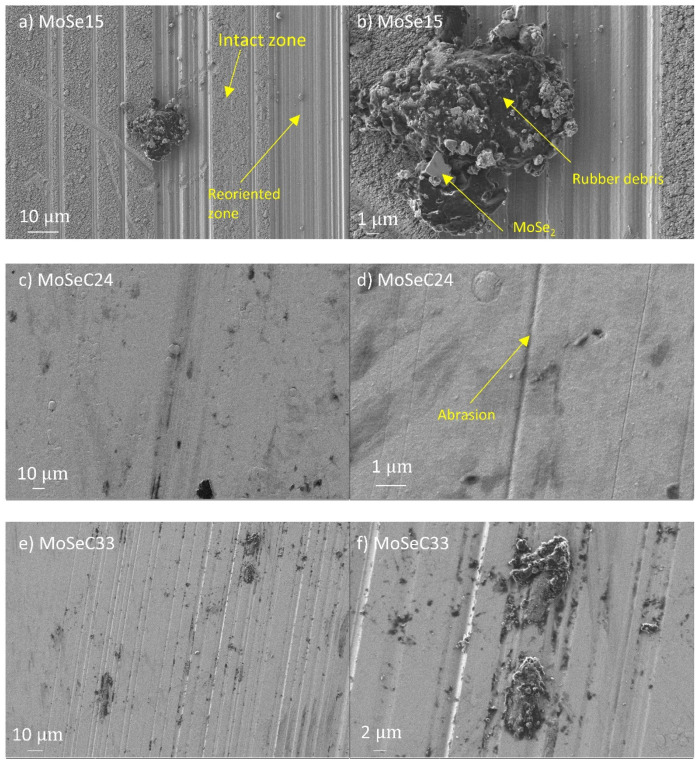

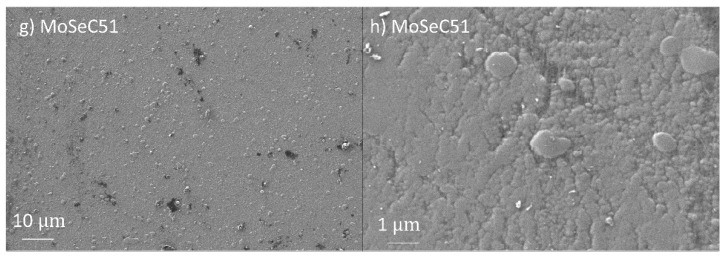
Micrographs of the wear tracks for the Mo-Se-C coatings after POD experiments against rubber at 25 °C: (**a**) MoSe at lower magnification (LM) imaging; (**b**) detailed features of the MoSe at high magnification (HM) imaging; (**c**) MoSeC24 at LM imaging; (**d**) detailed features of the MoSeC24 at HM imaging; (**e**) MoSeC33 at LM imaging; (**f**) MoSeC33 at HM imaging; (**g**) MoSeC51 at LM imaging; and (**h**) MoSeC51 at HM imaging.

As for the wear results, only the unalloyed MoSe coating revealed quantifiable wear (with SWR of ~1–2 × 10^−6^ mm^3^/Nm), while the alloyed films exhibited extremely little wear. The latter had wear depths of solely several nanometers, which is difficult to quantify. SEM imaging was performed to illustrate the morphological features of the wear scars. The micrographs of the wear tracks for the coatings are shown in Figure 10, with the left panel displaying lower magnification imaging, and the right panel revealing details through higher magnification imaging.

**Figure 11:**
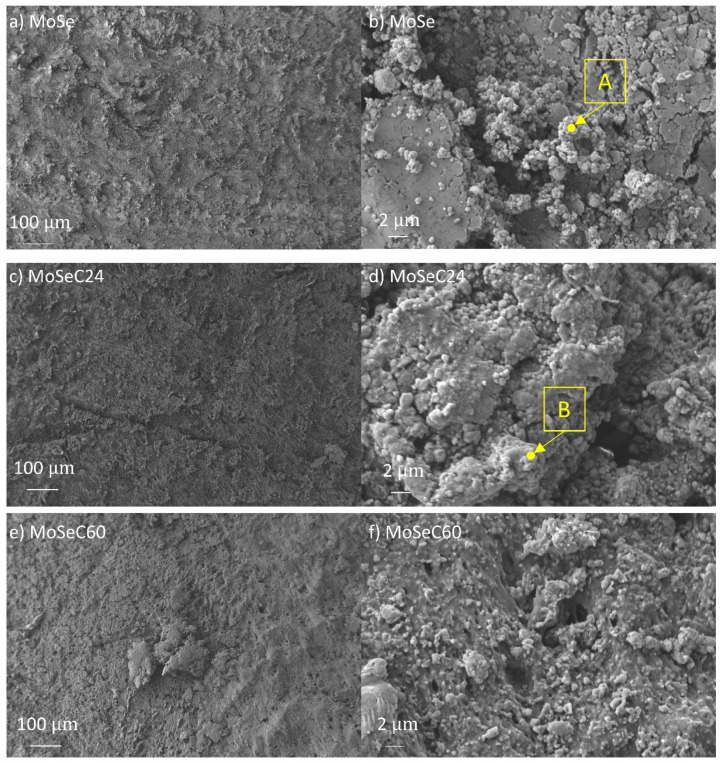
SEM micrographs of the rubber counterbodies, after performing the POD tests, at 25 °C: (**a**) sliding vs. MoSe coating, LM; (**b**) sliding vs. MoSe coating, HM details; (**c**) sliding vs. MoSeC24, LM; (**d**) sliding vs. MoSeC24, HM detail; (**e**) sliding vs. MoSeC60, LM; (**f**) sliding vs. MoSeC60, HM detail.

Figure 11 displays the SEM micrographs for the rubber counterbodies.

The wear track of the MoSe coating (see Figure 10a) exhibited areas which appeared intact (e.g., as-deposited state), and zones with a smoother appearance indicating the reorientation of the MoSe_2_ crystals. Based on 3D optical profilometry (not shown), these reoriented areas displayed wear depths up to 200 nm. Additionally, a very dark wear debris was present. To check this dark feature, EDS was performed. It detected a strong presence of carbon and small amounts of nitrogen, meaning that the debris originates from the rubber counterbody. The rubber debris intermixed with other material that had a greyish colour and smooth appearance, likely MoSe_2_. Overall, the rubber counterbody possessed a very rough and irregular appearance. EDS performed in some regions revealed Se and Mo presence (totalling up to 70 at. % in the area marked A in Figure 11b), indicating that material transfer existed from the coating to the counterbody. In the case of the alloyed films, the SEM imaging on the coatings revealed smoothening of the surface (compare, for instance, Figure 3h and Figure 10h), i.e., the cauliflower-like features flattened after the sliding test against the NBR counterbody. A morphological difference visualized between the wear track of the coatings with lower and higher carbon content was that abrasive marks were present in the former ones. Due to the inferior hardness and density of the films with lower carbon content, a detachment of material from the top-most part of the coating, which stayed at the NBR/coating interface, probably happened and caused abrasion. Furthermore, considering both that low carbon content coatings were richer in MoSe_2_ and had lower CoF than those with high carbon, lubricious MoSe_2_ was the most probable phase found at the interface. The previously mentioned material transfer phenomenon was confirmed via EDS analysis of the rubber counterbody, after testing against the MoSeC24 coating. For example, in the area marked as B in Figure 11d, a small amount of Se and Mo (totalling ~7 at. %) was detected. The coatings with a high carbon content (MoSeC51 and MoSeC60) had significantly less MoSe_2_ and, also, reduced crystallinity (see the GIXRD results). For these films to provide significant friction reduction, the crystallization and reorientation process of the TMD phase was required. However, due to the low contact stresses originating from the low hardness and elastic modulus of NBR, the conditions for crystallization of the TMD phase were not fully met. Hence, the lubricity provided was much lower. Additionally, due to the augmented difference in hardness between the higher carbon content coatings and the NBR ball, the wear rate was higher for the rubber counterbody. This manifested into more rubber material released at the interface, causing an increase in the COF. The EDS analysis of the NBR counterbody after the test against the MoSeC60 coating did not reveal any Se or Mo, supporting the aforementioned hypothesis.

#### 3.6.4. Sliding against Rubber (NBR) at High Temperature (200 °C)

**Figure 12:**
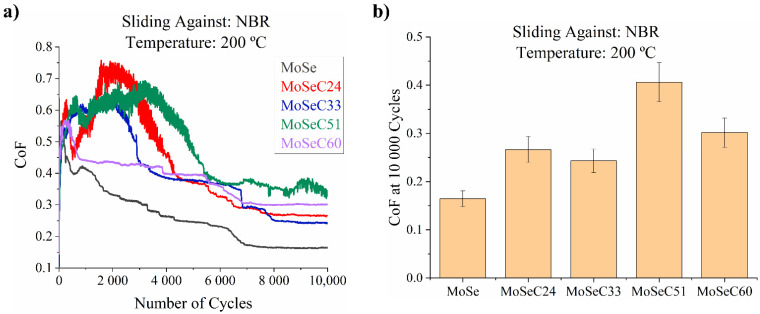
Results of the POD tests, performed against a NBR countersurface, at 200 °C: (**a**) friction curves; (**b**) final CoF.

**Figure 13:**
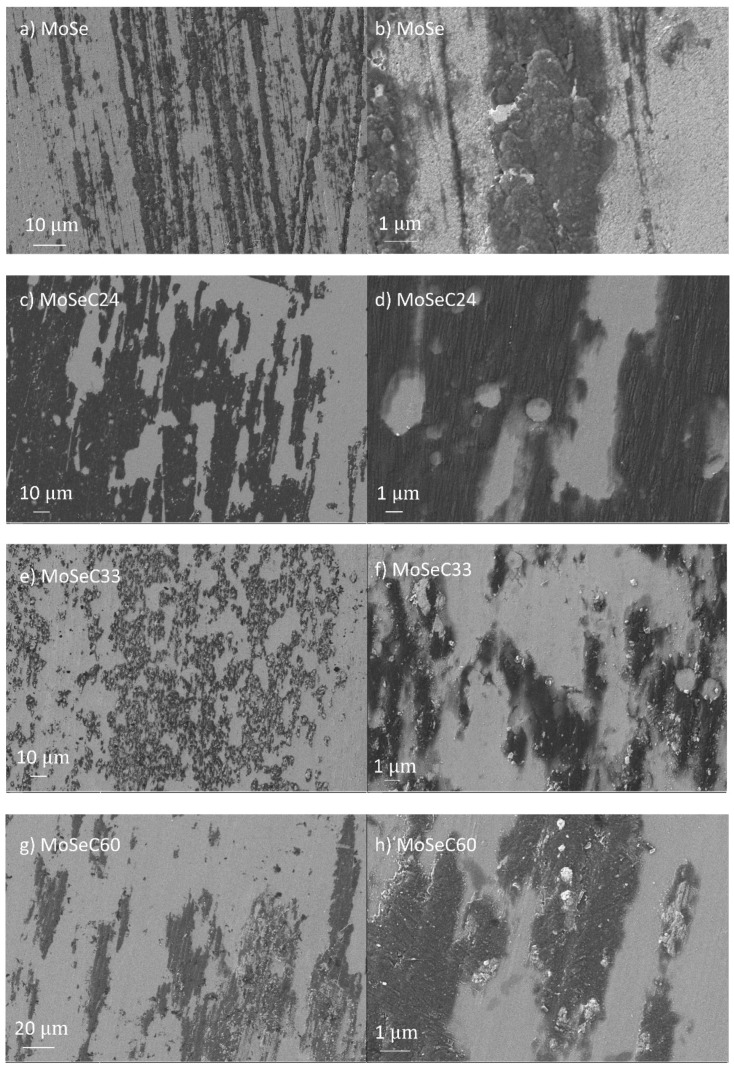
Micrographs of the wear tracks from the coatings, after sliding against rubber, at 200 °C: (**a**) MoSe at lower magnification (LM) imaging; (**b**) MoSe at high magnification (HM) imaging; (**c**) MoSeC24 at LM; (**d**) MoSeC24 at HM; (**e**) MoSeC33 at LM; (**f**) MoSeC33 at HM; (**g**) MoSeC60 at LM; (**h**) MoSeC60 at HM.

**Figure 14:**
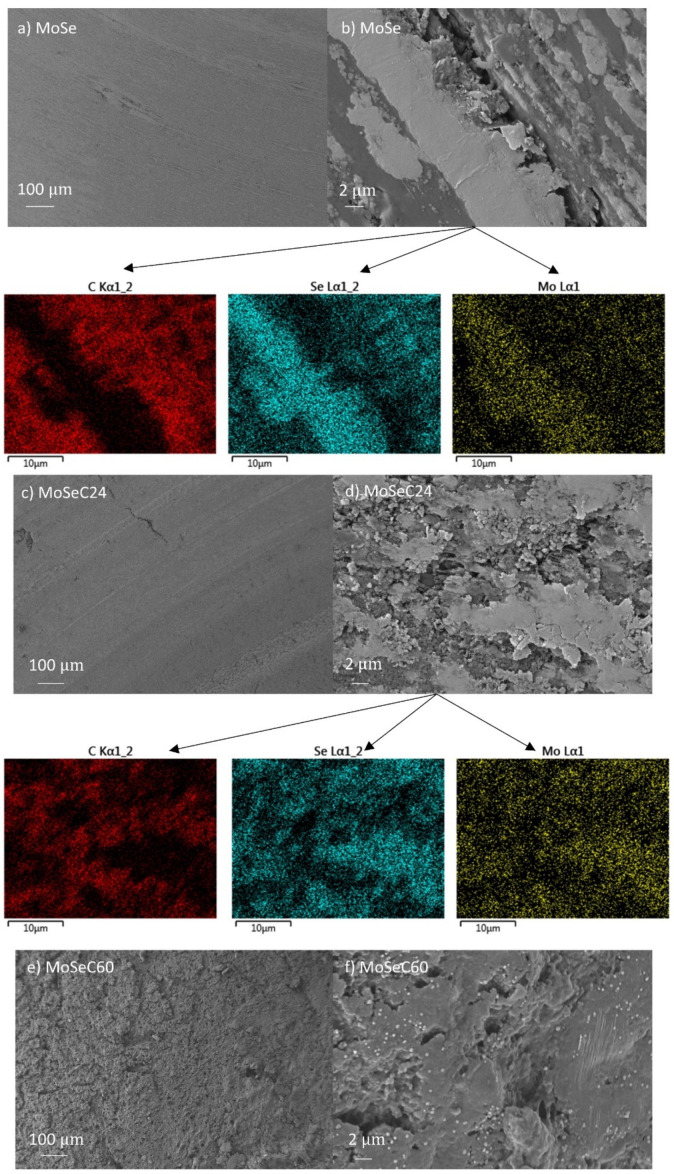
SEM/EDS of the rubber counterbodies performed after POD experiments at 200 °C: (**a**) for MoSe at LM; (**b**) for MoSe at HM; (**c**) for MoSeC24 at LM; (**d**) for MoSeC24 at HM; (**e**) for MoSeC60 at LM; (**f**) for MoSeC60 at HM.

Figure 12 displays the tribological results from the elevated temperature tests using NBR balls as counterbodies. Compared to the results obtained for ambient temperature testing, a reduction in the COF occurred. The best response was observed for the MoSe coatings, translating into a final COF of ~0.15–0.2. Afterwards, MoSeC24 and MoSeC33 demonstrated the most excellent performance, where values in the range of 0.25–0.3 were measured. Finally, the coatings with the highest carbon content (MoSeC51 and MoSeC60) were the worst performers. These had values of ~0.4 and ~0.3, respectively. Analogously to the results for ambient temperature tests, the coatings alloyed with less carbon presented improved performance than those doped with more carbon. Additionally, the wear was non-quantifiable for alloyed films. For the MoSe coatings, a SWR of ~1–2 × 10^−5^ mm^3^/Nm was measured. SEM/EDS analysis was carried on the wear tracks of the coatings and rubber counterbodies. The results are shown in Figure 13 and Figure 14, respectively.

The main distinction between the morphology of the wear tracks for the tests conducted at ambient and elevated temperature was the increased area covered with material displaying dark appearance. The EDS analysis indicated (it consisted of C and N) the presence of rubber, which probably stuck to the surface of the coatings. It is likely that the rubber properties were modified at high temperature. Unquestionably, the local temperature at the sliding region was substantially higher than that of the environment. Moreover, it was probably much superior to 200 °C in certain contacting spots on the rubber surface. Therefore, rubber likely underwent degradation in the presence of oxygen and frictional heating. In fact, in a study by Dong et al. [16], NBR underwent heat accumulation under dry sliding conditions, which led to the diminished tensile and tear strength of NBR, resulting in an increased average wear mass loss. In the present study, the rubber material transferred on the wear tracks seemed to smear along the sliding direction. Indeed, the unalloyed MoSe coating exhibited a smoother appearance relative to its as-deposited state, hinting to the reorientation of the MoSe_2_ crystals. Moreover, the NBR ball sliding against the MoSe coating at elevated temperature showed a smoother appearance than that tested at ambient temperature. Layers of material with a brighter appearance (see Figure 14b), smeared in the sliding direction, were observed. The EDS mapping performed indicated that these areas were rich in Mo and Se. The morphology of the carbon alloyed coatings also appeared smoother, whereas the NBR counterbody sliding against the MoSeC24 coating had smeared layers rich in Mo and Se (see Figure 14d and the respective maps). The NBR counterbody sliding against the coating with a higher carbon content (MoSeC60) did not reveal such details and generally displayed a rougher appearance compared to the NBR counterbodies sliding against the MoSe and MoSeC24 films (see Figure 14e). EDS analysis was performed in various spots for the MoSeC60 coating, and extremely small amounts of Se and Mo (<1 at. %) were detected, and thus no EDS mapping was performed. Globally, the reduction in friction verified during elevated temperature sliding against an NBR counterbody can be associated with the suggested mechanisms of improved shear properties of the MoSe_2_ phase in dry environments and facilitated crystallization of the MoSe_2_ phase. Furthermore, another mechanism should be considered in this case. With the increase in testing temperature, the rheology of the rubber material (the counterbody and the transferred rubber layers) is expected to decrease [40]. Therefore, it will be easier to shear, resulting in lower CoF. In this context, it should also be noted that a POD experiment of NBR ball sliding against an AISI M2 steel disk at 200 °C yielded a COF of ~0.6–0.7. The friction reduction due to the presence of MoSe_2_ on the sliding interface likely augments for coatings richer in MoSe_2_. Thus, the decreased CoF for the coatings with superior carbon content, at elevated temperature, should mainly be due to the different rheology of the rubber at these temperatures. The role of the carbon phase during the high-temperature sliding is again mainly towards the load support. Although elevated temperature can facilitate the graphitization of the carbon phase, hydrogen-free graphite is not an adequate solid lubricant in atmospheres lacking humidity. For example, sputtered hydrogen-free a-C coatings have deteriorated CoF as soon as the testing temperature increases [41].

## 4. Conclusions

In this work, we presented the synthesis of Mo-Se-(C) coatings with varied carbon contents, in semi-industrial conditions. This study sheds light on the compositional, microstructural and mechano-tribological properties. The main conclusions were:

- The Se/Mo ratio dropped with the increase in the carbon content, due to an increased bombardment of the growing film with Ar^+^ ions when the current applied to the graphite targets is increased.
- Even small additions of carbon (24 at. %) increased the compactness of the coating. Further additions of carbon resulted in a more compact morphological appearance, with decreased porosity and roughness.
- The coatings possessing carbon up to 33 at. % of C showed crystalline MoSe_2_ during XRD analysis. The films with higher carbon content (51 at. % and 60 at. %) were XRD amorphous. Generally, increased carbon contents negatively affected the crystallinity of the MoSe_2_ phase.
- The hardness correlated with the amount of the carbon phase of the coatings. The maximum value reached 7.4 GPa for the films with the highest carbon content.
- The frictional response aligned with the coating composition, since the alloyed coatings presenting a smaller amount of carbon exhibited lower CoF. Moreover, sliding against an NBR counterbody manifested into higher CoF as compared to the testing performed against a steel counterbody. Analogously, the CoF was lower when sliding at 200 °C, relative to the 25 °C experiments.

This work presented the potential of alloyed TMD coatings for friction and wear reduction against conventional steel and rubber-based counterbodies.

## Figures and Tables

**Table 1:** Composition of the steel samples used (in weight %).

Steel Type	C	Cr	Mo	W	V	Fe
AISI M2	0.85	4	5	6	2	Balance
DIN 100Cr6	1	1.5	<0.1	/	/	Balance

**Table 2:** Composition, roughness, thickness and deposition rate of the coatings based on WDS, AFM and SEM measurements, respectively.

Coatings	Composition (at. % of C)	Average Roughness (nm)	Thickness (µm)	Deposition Rate (nm/min)
MoSe	6	26.3	2.7	27
MoSeC24	24	7.2	1.2	12
MoSeC33	33	5.1	1.1	11
MoSeC51	51	4.6	1.3	13
MoSeC60	60	4.5	1.6	16

**Table 3:** Average critical load at which each failure event occurred during scratch test on the 5 distinct carbon content samples and HF grade attributed to each coating based on Rockwell C indentation.

Coatings	Lc1 [N]	Lc2 [N]	Lc3 [N]	HF Grade
MoSe	-	-	6.5 (±0.5)	6
MoSeC24	-	-	11 (±1.5)	5
MoSeC33	-	-	19 (±1)	4
MoSeC51	-	26 (±0.5)	>80 N	2
MoSeC60	38 (±1)	43 (±1)	>80 N	2

## Data Availability

The data presented in this study are available on request from the corresponding author.

## Nomenclature

**Symbol or Abbreviation**	**Full Name**
a-C	amorphous carbon
AFM	atomic force microscopy
AISI	American iron and steel institute
at. %	atomic percent
CoF	coefficient of friction
d	sliding distance [m]
DC	direct current
DIN	Deutsches Institut für Normung
DLC	diamond-like carbon
EDS	energy dispersive spectroscopy
E*	reduced Young’s modulus [Pa]
F	normal load [N]
FESEM	field emission scanning electron microscope
GDP	gross domestic product
GIXRD	grazing incidence X-ray diffraction
H	hardness [Pa]
Lc	critical load [N]
Mo-Se-C	molybdenum-selenium-carbon
MSPLD	magnetron-assisted pulsed laser deposition
MtCo_2_	megatons of CO_2_
NBR	nitrile butadiene rubber
POD	pin-on-disk
RF	radio frequency
RH	relative humidity
SEM	scanning electron microscope
SWR	specific wear rate
TMD	transition metal dichalcogenide
TMD-C	carbon alloyed transition metal dichalcogenide
TMDs	transition metal dichalcogenides
VDI	verein deustcher ingenieure
Vw	worn volume [mm^3^]
WDS	wavelength dispersive spectroscopy
W-S-C	tungsten-sulphur-carbon
Wr	specific wear rate [mm^3^/Nm]
